# Supplementary material for: Synthesis and systematic review of reported neonatal SARS-CoV-2 infections
Source: Nat Commun. 2020 Oct 15;11:5164. doi: 10.1038/s41467-020-18982-9 (PMC7566441; doi:10.1038/s41467-020-18982-9)
Supplement: Supplementary file 2 — Reporting Summary [file 41467_2020_18982_MOESM2_ESM.pdf]

nature research

Corresponding author(s): Daniele De Luca

Last updated by author(s): Sep 11, 2020

Reporting Summary

Nature Research wishes to improve the reproducibility of the work that we publish. This form provides structure for consistency and transparency in reporting. For further information on Nature Research policies, see our Editorial Policies and the Editorial Policy Checklist.

Statistics

For all statistical analyses, confirm that the following items are present in the figure legend, table legend, main text, or Methods section.

n/a	Confirmed	
☐	☒	The exact sample size (*n*) for each experimental group/condition, given as a discrete number and unit of measurement
☒	☐	A statement on whether measurements were taken from distinct samples or whether the same sample was measured repeatedly
☐	☒	The statistical test(s) used AND whether they are one- or two-sided<br>*Only common tests should be described solely by name; describe more complex techniques in the Methods section.*
☐	☒	A description of all covariates tested
☐	☒	A description of any assumptions or corrections, such as tests of normality and adjustment for multiple comparisons
☐	☒	A full description of the statistical parameters including central tendency (e.g. means) or other basic estimates (e.g. regression coefficient) AND variation (e.g. standard deviation) or associated estimates of uncertainty (e.g. confidence intervals)
☒	☐	For null hypothesis testing, the test statistic (e.g. *F*, *t*, *r*) with confidence intervals, effect sizes, degrees of freedom and *P* value noted<br>*Give P values as exact values whenever suitable.*
☒	☐	For Bayesian analysis, information on the choice of priors and Markov chain Monte Carlo settings
☒	☐	For hierarchical and complex designs, identification of the appropriate level for tests and full reporting of outcomes
☒	☐	Estimates of effect sizes (e.g. Cohen's *d*, Pearson's *r*), indicating how they were calculated

Our web collection on statistics for biologists contains articles on many of the points above.

Software and code

Policy information about availability of computer code

Data collection	Excel 16 (Microsoft Corporation, Redmond, WA-USA) and MedCalc 13.3 (MedCalc, Ostend, Belgium) and Zotero (5.0.87, Roy Rosenzweig Centre for History and New Media, Fairfax, VI – USA)
Data analysis	Excel 16 (Microsoft Corporation, Redmond, WA-USA) and MedCalc 13.3 (MedCalc, Ostend, Belgium) and Mayo Evidence-Based Practice Centre tool (as described in Murad, M. H., Sultan, S., Haffar, S. & Bazerbachi, F. Methodological quality and synthesis of case series and case reports. BMJ Evid.-Based Med. 23, 60–63 (2018).)

For manuscripts utilizing custom algorithms or software that are central to the research but not yet described in published literature, software must be made available to editors and reviewers. We strongly encourage code deposition in a community repository (e.g. GitHub). See the Nature Research guidelines for submitting code & software for further information.

Data

Policy information about availability of data

All manuscripts must include a data availability statement. This statement should provide the following information, where applicable:

- Accession codes, unique identifiers, or web links for publicly available datasets
- A list of figures that have associated raw data
- A description of any restrictions on data availability

All summary data generated during this study are included in this published article. Raw data used for the analyses are available upon request or presented in the original reviewed articles which have been retrieved from the following publicly available databases: PubMed (http://www.ncbi.nlm.nih.gov/pubmed/), The Cochrane Library (https://www.cochranelibrary.com), Web of Science (https://clarivate.com/webofsciencegroup/solutions/web-of-science/), BioXRiv (https://www.biorxiv.org) and MedXRiv (https://www.medrxiv.org).

Field-specific reporting

Please select the one below that is the best fit for your research. If you are not sure, read the appropriate sections before making your selection.

- [x] Life sciences
- [ ] Behavioural & social sciences
- [ ] Ecological, evolutionary & environmental sciences

For a reference copy of the document with all sections, see nature.com/documents/nr-reporting-summary-flat.pdf

Life sciences study design

All studies must disclose on these points even when the disclosure is negative.

Sample size	N.A. because it is a meta-analysis. We used all the electronic article databses tipically used in any meta-analyses. there are no other medical article database that are commonly used to this end
Data exclusions	We included only articles describing neonates infected by SARS-CoV-2 (i.e. infants within the first month of life), as demonstrated by: 1) at least one positive real-time reverse transcription polymerase chain reaction (RT-PCR) on nasopharyngeal swabs, and/or 2) positive serology with detection of specific IgM. Articles were excluded according to pre-defined criteria as usual for meta-analyses and systematic reviews. These criteria were the following: conference abstracts, any report of neonates exposed to but not infected by SARS-CoV-2 (according to the aforementioned criteria), articles describing only stillbirths or children aged more than 30 days and manuscript reporting hypotheses or opinions without any clinical data. Duplicate reports and "grey" literature were also excluded. Excluded articles and reasons for exclusion are detailed in fig.1 in the manuscript.
Replication	N.A. because it is a meta-analysis. Replication makes no sense here: hus it is just a "desk analysis" of published cases based on simple arythmetics. If you redo the calculation, without changing the meta-analysed articles, you will always get the same reuslts.
Randomization	N.A. because it is a meta-analysis. Randomization makes no sense here: Randomization is a technique needed in clinical trials. It is not needed in meta-analysis and systenatic reviews., as there are no patients potentially receiving one treatment/test or the other. See also above and PRISMA guidelines which do not mention any need for randomization.
Blinding	N.A. because it is a meta-analysis. Blinding makes no sense here: blinding is a technique for clinical trials and ours is not a clinical trial. We have no patients to randomise to a treatment/test or another and no outcome to mask.

Reporting for specific materials, systems and methods

We require information from authors about some types of materials, experimental systems and methods used in many studies. Here, indicate whether each material, system or method listed is relevant to your study. If you are not sure if a list item applies to your research, read the appropriate section before selecting a response.

Materials & experimental systems

n/a	Involved in the study	
x		Antibodies
x		Eukaryotic cell lines
x		Palaeontology and archaeology
x		Animals and other organisms
	x	Human research participants
x		Clinical data
x		Dual use research of concern

Methods

n/a	Involved in the study	
x		ChIP-seq
x		Flow cytometry
x		MRI-based neuroimaging

Human research participants

Policy information about studies involving human research participants

Population characteristics	neonates, that is infants from 0 to 30 days of age, of any sex, from any type of delivery, infected by SARS-CoV-2 (i.e. infants within the first month of life), as demonstrated by: 1) at least one positive real-time reverse transcription polymerase chain reaction (RT-PCR) on nasopharyngeal swabs, and/or 2) positive serology with detection of specific IgM.
Recruitment	We looked for cohort, cross-sectional and case-control studies, as well as case series or case reports published as articles or letters to the editors on PubMed, The Cochrane Library, Web of Science, as well as BioXRiv and MedXRiv preprint archives. We used the following keywords or MeSH terms: "Coronavirus", "COVID-19", "SARS-CoV-2", "newborn", "preterm" and "neonates". We also hand-searched references cited in the eligible manuscripts or in review articles on the subject and the authors' personal archives. We used the following Boolean string: (COVID-19 AND neonates) OR (coronavirus AND newborn)) OR (coronavirus AND preterm)) OR (coronavirus AND neonates)) OR (SARS-CoV-2 AND newborn)) OR (SARS-CoV-2 AND preterm)) OR (SARS-CoV-2 AND neonates)) OR (COVID-19 AND preterm)) OR (COVID-19 AND newborn)) AND (("2019/12/01"[Date - Create] : "2020/07/14"[Date - Create])).<br>POSSIBLE biases are the publication bias as not all neonatal SARS-CoV-2 infections get published in the medical literature and this may have reduced our analysed population. Another possible bias is the lack of detail in every single meta-analysed article that may have reduced the precision. However articles' quality has been evaluated in a known and standardised way. Biases are detailed at the end of discussion, as study limitations, in the manuscript
Ethics oversight	not needed as it is a meta-analysis. The French Ethical Committee for the Research in Obstetrics and Gynecology reviewed the work and confirmed that the institutional review board approval was unnecessary. See attached

Note that full information on the approval of the study protocol must also be provided in the manuscript.
